# Lack of fit with the neighbourhood social environment as a risk factor for psychosis – a national cohort study

**DOI:** 10.1017/S0033291721002233

**Published:** 2023-02

**Authors:** Peter Schofield, Jayati Das-Munshi, Roger T. Webb, Henriette Thisted Horsdal, Carsten B. Pedersen, Esben Agerbo

**Affiliations:** 1 School of Population Health & Environmental Sciences, Faculty of Life Sciences & Medicine, King's College London, London, UK; 2 ESRC Centre for Society and Mental Health, King's College London, London, UK; 3 Institute of Psychiatry, Psychology & Neuroscience, King's College London, London, UK; 4 South London & Maudsley NHS Foundation Trust, London, UK; 5 Division of Psychology and Mental Health, Faculty of Biology, Medicine and Health, The University of Manchester, Manchester Academic Health Sciences Centre (MAHSC), Manchester, UK; 6 National Institute of Health Research (NIHR) Greater Manchester Patient Safety Centre, Manchester, UK; 7 National Centre for Register-Based Research (NCCR), Aarhus University, Aarhus, Denmark; 8 CIRRAU – Centre for Integrated Register-based Research at Aarhus University, Aarhus, Denmark

**Keywords:** Etiology, social determinants, psychosis, schizophrenia, ethnic density, minority groups

## Abstract

**Background:**

Many studies report an ethnic density effect whereby psychosis incidence among ethnic minority groups is higher in low co-ethnic density areas. It is unclear whether an equivalent density effect applies with other types of socioeconomic disadvantages.

**Methods:**

We followed a population cohort of 2 million native Danes comprising all those born on 1st January 1965, or later, living in Denmark on their 15th birthday. Socioeconomic disadvantage, based on parents' circumstances at age 15 (low income, manual occupation, single parent and unemployed), was measured alongside neighbourhood prevalence of these indices.

**Results:**

Each indicator was associated with a higher incidence of non-affective psychosis which remained the same, or was slightly reduced, if neighbourhood levels of disadvantage were lower. For example, for individuals from a low-income background there was no difference in incidence for those living in areas where a low-income was least common [incidence rate ratio (IRR) 1.01; 95% confidence interval (CI) 0.93–1.10 *v.* those in the quintile where a low income was most common. Typically, differences associated with area-level disadvantage were the same whether or not cohort members had a disadvantaged background; for instance, for those from a manual occupation background, incidence was lower in the quintile where this was least *v.* most common (IRR 0.83; 95% CI 0.71–0.97), as it was for those from a non-manual background (IRR 0.77; 95% CI 0.67–0.87).

**Conclusion:**

We found little evidence for group density effects in contrast to previous ethnic density studies. Further research is needed with equivalent investigations in other countries to see if similar patterns are observed.

## Background

Disadvantaged minority status is associated with an increased incidence of psychosis, with membership of a minority ethnic group being the most often reported example (Selten, Van Der Ven, & Termorshuizen, 2019). In Western European countries, incidence is typically highest among those whose origins are in low- and middle-income countries, and among Black ethnic minority groups in particular (Bourque, van der Ven, & Malla, 2011; Cantor-Graae & Selten, 2005). Conversely, elevated psychosis incidence is not apparent where migrant groups do not constitute a numerical minority (Corcoran et al., 2009). This is clearly seen at the neighbourhood level where studies consistently show that the extent to which members of an ethnic group are in a minority, i.e. their neighbourhood ethnic density, is inversely related to their psychosis risk (Bécares, Dewey, & Das-Munshi, 2018; Shaw et al., 2012). Some proposed explanations for this effect are specific to ethnicity; for example, that neighbourhood ethnic density has a buffering effect against racial discrimination (Becares, Cormack, & Harris, 2013; Bécares et al., 2009; Das-Munshi, Becares, Dewey, Stansfeld, & Prince, 2010; 2012). However, other more general explanations are also proposed; for example, that this reflects more general improvements in social support, as a result of living with others with shared circumstances (Das-Munshi et al., 2010, 2012; Shaw et al., 2012). This may in turn reflect more fundamental aspects of a shared lifestyle, or ‘habitus’, and a sense of localised identity having a positive effect on overall psychological wellbeing (Bourdieu, 1984; Halpern, 1993). Conversely, it is argued, a lack of fit with the neighbourhood social environment can lead to social marginalisation and a state of social ‘defeat’ associated with psychosis (Gevonden et al., 2014; Selten et al., 2001; Selten & Cantor-Graae, 2005; Zammit et al., 2010).

Should these more general explanations apply then we might expect to see a similar density effect for other indices of disadvantage. In recent years, however, the focus of minority density studies has been on ethnicity alone. There is some, albeit limited, evidence that being in a minority in a neighbourhood due to one's relatively low socioeconomic position is associated with elevated suicide risk (Platt, 1986; Schofield et al., 2016), although a national Danish register-based study did not find such a pattern (Agerbo, Sterne, & Gunnell, 2007). Other studies, however, found a relationship of this nature with depression (Albor et al., 2014) and rates of psychiatric hospitalisation (Wechsler & Pugh, 1967). The latter US study was the first to explicitly set out to test the ‘fit’ hypothesis; that people with a particular personal characteristic living in communities where that characteristic is less common should have a higher rate of psychiatric hospitalisation. Others have found that being in a minority due to one's sexuality (Hatzenbuehler, Keyes, & McLaughlin, 2011) and religion (Rosenberg, 1962) are also risk factors for psychiatric morbidity. However, all these studies rely on cross-sectional data making it difficult to rule out social drift as a possible alternative explanation (Dohrenwend et al., 1992). This could apply both where illness onset results in a move to a different neighbourhood, as well as instances where someone's social circumstances change due to illness, so that they become incongruent with their neighbourhood's norms (Marwaha et al., 2007; Meltzer et al., 2002). Ideally area-level exposures should therefore be measured well in advance of disease onset and risk factors should be independent of any predisposition, including parental determinants, towards developing a mental disorder. To our knowledge only one study has investigated this issue using longitudinal data. This Swedish population-based study examined individual, school and municipality levels (Zammit et al., 2010) and reported school level density effects using composite measures of deprivation and social fragmentation. However, it is less clear how the latter index could be interpreted at an individual level and, as this includes migrant status as a component, it is difficult to distinguish the effect of social fragmentation from that of ethnic density.

In the current study, we assessed a range of specific social disadvantage indices using whole population data to determine whether associations with psychosis incidence are modified by whether the disadvantage is common or rare in a person's neighbourhood. We looked at different types of disadvantages reported as being associated with psychosis in previous studies (Byrne, Agerbo, Eaton, & Mortensen, 2004; Corcoran et al., 2009; Eaton, 1974; Hakulinen, Webb, Pedersen, Agerbo, & Mok, 2020; Morgan et al., 2008; Muntaner, Tien, Eaton, & Garrison, 1991; Werner, Malaspina, & Rabinowitz, 2007), with each measured at the parental level to preclude reverse causality. These measures included indices of socioeconomic disadvantage (parental non-employment and low income), occupational social class (manual *v.* non-manual) and social fragmentation (single-parent family status). Comparing density effects for a range of different types of social disadvantages, we aimed to explicate potential mechanisms that could plausibly explain observed contextual effects.

We therefore assessed whether associations between indices of disadvantage at age 15 and later onset of psychosis are modified according to the prevalence of the same disadvantage in a person's neighbourhood. We followed a similar methodology to previous ethnic density studies; our hypothesis being that, for socioeconomic disadvantages, equivalent density effects exist.

## Method

### Sample

As with our two previous ethnic density studies (Schofield et al., 2017, 2018) we followed a population cohort comprising all persons whose 15th birthday came after 1st January 1980 and before 31st December 2012. Cohort members were followed up from their 15th birthdays until they died, emigrated, were diagnosed with a non-affective psychosis or 1st July 2013, whichever came first. We utilised data collected under the Danish Civil Registration System, which enabled a range of population registers to be interlinked using the unique personal identification numbers that are assigned to all Danish citizens (Pedersen, 2011).

### Outcomes

From the Danish Psychiatric Central Register, which covers all psychiatric in-patient admissions and, since 1995, all out-patient visits (Mors, Perto, & Mortensen, 2011), incident cases of non-affective psychosis (i.e. schizophrenia or related disorders) were ascertained according to discharge diagnosis using ICD-10 codes: F20–29; or equivalent ICD-8 codes: ICD-8 295.x9, 296.89, 297.x9, 298.29–298.99, 299.04, 299.05, 299.09 and 301.83. Date of onset was defined as the date of first contact with this diagnosis (whether as an inpatient, outpatient or through an emergency psychiatric care unit).

### Exposures

We examined each specific indicator of disadvantaged status at the individual and neighbourhood levels, with both determined when the cohort member was aged 15. Local neighbourhood was defined using small area units derived from Danish parishes, median population size 3564, which we adapted for our previously conducted ethnic density studies (Schofield et al., 2017, 2018).

Each type of disadvantage was measured at the individual and neighbourhood levels, in the year of the cohort member's 15th birthday, as follows:

1. Employment status: at the individual level, was defined as father not employed (*v.* father employed). Neighbourhood job status profile was defined as the proportion of males of working age who were not employed (excluding students).
2. Occupational status: father having an ‘other manual/elementary occupation’ – based on the International Standard Classification of Occupations (ISOC, 08). Neighbourhood occupational profile was defined as the proportion of males of working age with this level of occupation. Area-level occupational data were only available from 1991.
3. Family structure: mother's marital status being single (not cohabiting). At the neighbourhood level this was defined as the proportion of single mothers in the neighbourhood, following the same definition.
4. Family income: low income based on the combined income of both parents (or mother alone if single parent with missing data for father's income). A low income was defined as a combined income below the 25th centile, for that year, for families of the cohort member's peer group (i.e. those with 15-year-old children). We did not have access to information about the whole population's area-level proportion with a low income, and we therefore relied on information from within our cohort for this part of the analysis. Neighbourhood income profile was defined as the proportion of cohort members' peers, i.e. those aged 15, in the local area with parental (combined) income below the 25th centile. Restricting the population in this way meant that, for some neighbourhoods, the sample size was very low. Therefore, to enhance statistical power, for this part of the analysis only we combined data for the previous and subsequent 5 years.

### Covariates

We adjusted for age and gender (including age–gender interactions) and period effects as potential confounders. We also adjusted for neighbourhood urbanicity as incidence of psychosis has been shown to be raised in more heavily populated areas (Krabbendam & van Os, 2005; Pedersen, 2006; Vassos, Pedersen, Murray, Collier, & Lewis, 2012) in Northern Europe. Given the demographically mixed and socially fragmented nature of many urban areas we may expect that individuals in such environments are more likely to experience being in a minority socio-demographic group; therefore, urbanicity would have a confounding effect in our study. Urbanicity was derived at the parish level based on the locality's population density (residents per km^2^) in the year when the cohort member was 15, as applied in previously reported studies that were conducted using these registry data (Pedersen & Mortensen, 2001; Schofield et al., 2017). Some comparable ethnic density studies also adjust for neighbourhood deprivation (Kirkbride et al., 2007a; Veling et al., 2008b). In our study we are, in effect, already modelling area-level disadvantage and therefore would not expect this to make a material difference. To test this assumption, we included a sensitivity analysis adjusting for area-level income in the three models that do not themselves already include area-level income (see online Supplementary appendix Table 7). This made no appreciable difference to our results therefore we did not include this adjustment in the final model presented here. We also adjusted for parental psychiatric morbidity as this may influence the type of neighbourhood in which cohort members live at age 15, as well as being related to their own risk of subsequently developing psychosis. We adjusted for history of any psychiatric disorder in either parent prior to the cohort members' 15th birthdays as the clearest available indicator of relevant parental morbidity (Mors et al., 2011)

### Statistical analysis

We fitted multilevel Poisson regression models to estimate incidence rate ratios (IRRs) for non-affective psychosis, modelling the relationship with neighbourhood incongruence as a cross-level interaction between individual-level disadvantage and the proportion of people experiencing the same disadvantage in the local neighbourhood. For example, where being disadvantaged at age 15 was defined as ‘father not employed’, this was entered as an interaction with the neighbourhood job status profile, based on the proportion of working age males in the locality who were not employed.

We present two alternative models to give a more complete picture of neighbourhood effects: the first showing the effects of different levels of neighbourhood disadvantage for those in the relevant disadvantaged group and the second showing neighbourhood effects for those without the disadvantage.

To account for potential non-linearity, area-level proportions were modelled as categorical variables grouped into quintiles. In this way our analysis was directly comparable to our own previous ethnic density studies, also carried out in a Danish context (Schofield et al., 2017, 2018), as well as other previous ethnic density studies (Boydell et al., 2001; Veling, Hoek, & Mackenbach, 2008a, 2008b). In order to compare our results with those based on a linear model using a continuous exposure measure (Bécares et al., 2018) we also carried out the analysis using this alternative parameterisation and we present these results in the online Supplementary appendix (Tables 5 and 6). We also incorporated changing neighbourhood characteristics over time, including an extra level for year of exposure i.e. the multilevel model comprised of three levels: level 1: individuals; level 2: year of exposure (when aged 15) and level 3: neighbourhood. The exposure year (level 2) was included to account for changing neighbourhood indices over time.

We used the Lexis expansion method to incorporate age and period effects (calendar time) as time-varying covariates (Carstensen, 2007). Age was categorised in 5-year bands from 15–20 through 55 or older. Calendar time was categorised into 5-year bands, and 2-year bands in the 1990s, to account for changes as Denmark transitioned from the ICD-8 to the ICD-10 diagnostic system.

We excluded all migrant groups in order to rule out potential confounding between ethnic density and other group density effects. We excluded both first-generation (born outside Denmark) and second-generation (parents born outside Denmark) migrants using the definitions adopted previously (Schofield et al., 2017, 2018).

We found no evidence for over-dispersion in the fitted Poisson regression models (Breslow, 1984). All analyses were conducted using Stata software (version 15).

### Missing data

To account for missing data, for each indicator of disadvantage the denominator was the total number of people in the relevant population in a neighbourhood with complete data for that indicator. One exception was the neighbourhood proportion of single parents, which was unavailable at the area level. From the individual data it is apparent that missing marital status information is rare, with this occurring for only 1.3% of cohort members (see online Supplementary appendix Table 4). Therefore, this is unlikely to have made any material difference to the accuracy of our measure. Manual occupation was only recorded from 1991 and there was a large amount of missing data for the period from 2003 to 2006 due to a change in coding for this variable (~26% each year). Therefore, we have excluded these years from the occupational status analysis.

### Ethical approval

Ethical approval is not required to use the Danish registry data. However, access to use the data required formal approvals from the Danish Data Protection Agency, the Danish National Board of Health and Statistics Denmark.

## Results

In this national cohort of 1 924 607 people, aged 15 between 1980 and 2012, a total of 24 444 (1.3%) were diagnosed with a non-affective psychosis over 33.8 million person-years of follow-up.

### Disadvantaged status

Prevalence values for indices of disadvantage for this cohort at age 15 are shown in Table 1; for example, coming from a low-income family (22%), one where the father had a manual occupation (21%) and a single-parent family (17%). Not being employed was least common, with only 10% of cohort members coming from a family where the father was not employed when they were aged 15.

**Table 1.** IRRs of non-affective psychosis by disadvantaged status (at age 15)

Disadvantaged status (parent circumstances at age 15)	Total (*N*)	Percentage	Person-years	Cases	Crude incidence rate^a^	IRRs (95% CI)^b^
Not employed^c^	183 821	9.96	3 141 053	4253	13.54	1.71 (1.65–1.77)
Manual occupation^c^	188 259	20.72	1 859 061	2595	13.96	1.43 (1.37–1.50)
Single parent^d^	331 489	17.47	5 278 385	6953	13.17	1.75 (1.70–1.80)
Low income^e^	407 835	21.65	7 469 017	8316	11.13	1.63 (1.58–1.67)

a Number of new cases per 10 000 person-years at risk.

b Incidence rate compared with rate for cohort members without specified parental circumstances. All ratios were adjusted for age, gender, calendar period and any parental psychiatric history prior to age 15.

c Father's status.

d Mother only.

e Combined parents' gross income.

At the neighbourhood level there was wide variation in the proportion of people with each type of disadvantage (see Table 2). For example, for low-income status, in neighbourhoods where this was least common (lowest quintile), 15% were on a low income; rising to 43% in the highest quintile. Other types of disadvantages were similarly distributed although the overall proportion of people with a manual occupation background was greater.

**Table 2.** IRRs of non-affective psychosis by neighbourhood congruency (based on neighbourhood profile at age 15) for cohort members with each type of disadvantaged status

Disadvantaged status (parent circumstances at age 15) and corresponding neighbourhood profile (quintiles)	Average (mean) neighbourhood percentage with corresponding disadvantaged status (%)	Cases	Crude incidence rate^a^	IRRs (95% CI) – fully adjusted model^b^
Not employed				
1 (least common)	13	939	10.27	0.97 (0.88–1.08)
2	18	953	13.61	1.08 (0.97–1.20)
3	22	837	14.48	1.02 (0.92–1.13)
4	25	779	15.28	0.98 (0.89–1.09)
5 (most common)	32	745	16.97	1.00 (ref)
Manual occupation				
1 (least common)	24	743	11.37	0.83 (0.71–0.97)
2	32	685	12.46	0.81 (0.70–0.95)
3	38	495	16.80	0.95 (0.81–1.10)
4	43	396	18.61	1.01 (0.86–1.18)
5 (most common)	50	276	18.63	1.00 (ref)
Single parent				
1 (least common)	14	1416	10.50	0.78 (0.70–0.86)
2	21	1315	12.14	0.80 (0.72–0.88)
3	27	1394	13.37	0.87 (0.79–0.95)
4	34	1327	14.23	0.90 (0.83–0.98)
5 (most common)	44	1501	17.24	1.00 (ref)
Low income				
1 (least common)	15	1491	10.62	1.01 (0.93–1.10)
2	22	1756	11.21	1.10 (1.01–1.19)
3	26	1770	10.60	1.06 (0.98–1.15)
4	32	1815	10.53	1.06 (0.98–1.14)
5 (most common)	43	1484	13.43	1.00 (ref)

a The incidence rate measures the number of new cases per 10 000 person-years at risk.

b Adjusted for age, gender, calendar period, parental psychiatric history and neighbourhood urbanicity (quintile) at age 15.

At the individual level each measure of disadvantage was associated with an increased incidence of subsequent non-affective psychosis (Table 1). This association was strongest where the cohort member had a single-parent family background; with a 75% increased incidence, IRR 1.75 [95% confidence interval (CI) 1.70–1.80]; and weakest for those with a manual occupation background, IRR 1.43 (95% CI 1.37–1.50).

### Individual *v.* neighbourhood incongruence

For members of each disadvantaged group we compared the incidence of non-affective psychosis across neighbourhood type, based on the neighbourhood proportion with that same disadvantage. These were divided into quintiles and the comparisons presented here are made with reference to the most disadvantaged quintile. Our analysis using a continuous exposure measure yielded very similar results and these are presented in a separate online Supplementary appendix (Tables 5 and 6).

After adjusting for the above covariates, we found that there was typically little difference in the incidence of non-affective psychosis whether disadvantaged cohort members lived in neighbourhoods with a relatively high or low prevalence of the same disadvantage indicator. For example, there was no statistically significant difference found when comparing high and low disadvantage quintiles for: non-employment status, IRR 0.97 (95% CI 0.88–1.08); and low-income status, IRR 1.01 (95% CI 0.93–1.10). For those with a manual occupation and a single-parent family background, being in a neighbourhood where this disadvantage was less usual appeared to have a protective effect. There was a lower subsequent incidence of psychosis, IRR 0.83 (95% CI 0.71–0.97) and 0.78 (95% CI 0.70–0.86) for these groups in areas where manual occupation and single-parent families, respectively, were least prevalent compared to quintiles where they were most prevalent.

**Fig. 1.**
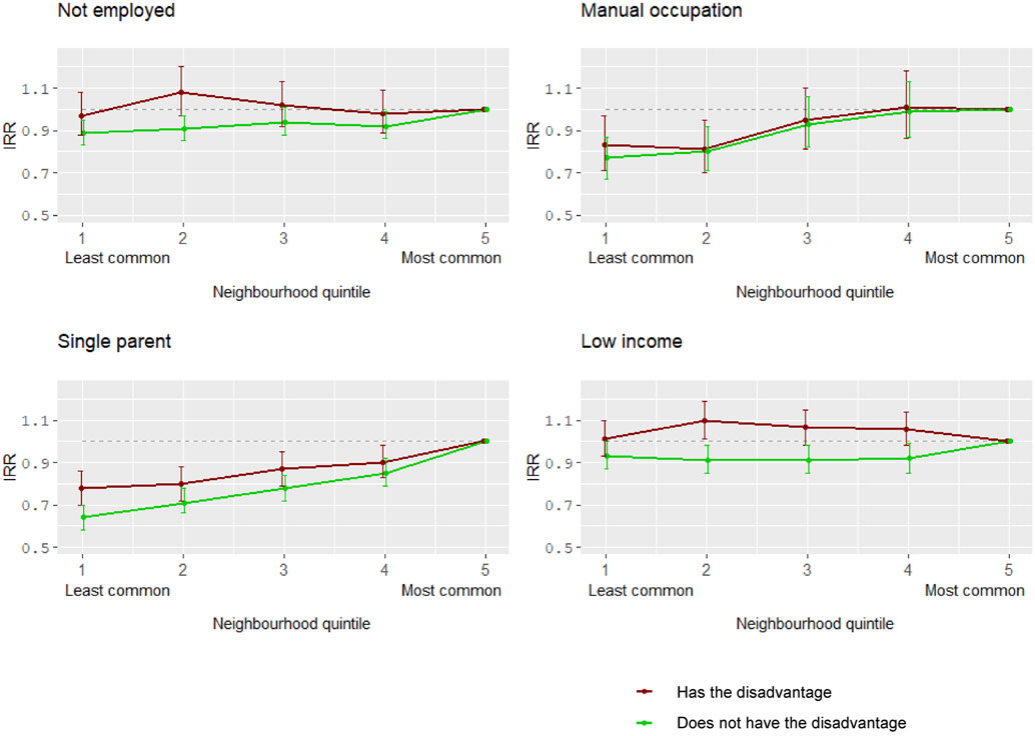
Association between psychosis incidence and neighbourhood congruence for different types of disadvantages – for those with and without the disadvantage.

We also carried out a similar analysis, this time looking at those without the corresponding disadvantage. For this group, living in an area with a lower level of disadvantage was typically associated with a reduced rate of psychosis (see Table 3). For example, for those who were employed, living in an area with the lowest proportion of non-employed was associated with a lower incidence of psychosis, IRR 0.89 (95% CI 0.83–0.95) compared to localities where this proportion was high. However, these differences were small and, in almost all instances, CIs overlapped with the corresponding neighbourhood effects for the disadvantaged group (this can be most clearly seen in Fig. 1). The one exception can be seen when comparing groups in the second highest income quintile although in the highest quintile risk ratios again appear to converge.

**Table 3.** IRRs of non-affective psychosis by neighbourhood congruency (based on neighbourhood profile at age 15) for cohort members without the corresponding disadvantaged status

Disadvantaged status *not present* (parent circumstances at age 15) and corresponding neighbourhood profile (quintiles)	Average (mean) neighbourhood percentage who do not have the corresponding disadvantaged status (%)	Cases	Crude incidence rate^a^	IRRs (95% CI) – fully adjusted model^b^
Not employed				
1 (least common)	87	6465	5.15	0.89 (0.83–0.95)
2	82	4355	6.35	0.91 (0.85–0.97)
3	78	3373	7.29	0.94 (0.88–1.01)
4	75	2527	7.65	0.92 (0.86–0.99)
5 (most common)	68	1722	8.96	1.00 (ref)
Manual occupation				
1 (least common)	76	3228	6.92	0.77 (0.67–0.87)
2	68	2000	8.04	0.80 (0.71–0.92)
3	62	960	10.85	0.93 (0.82–1.06)
4	57	610	11.91	0.99 (0.87–1.13)
5 (most common)	50	343	11.79	1.00 (ref)
Single parent				
1 (least common)	86	6526	4.84	0.64 (0.58–0.70)
2	79	3847	6.22	0.71 (0.66–0.78)
3	73	2880	6.94	0.78 (0.72–0.84)
4	66	2159	7.84	0.85 (0.79–0.92)
5 (most common)	56	1508	10.04	1.00 (ref)
Low income				
1 (least common)	85	5310	5.92	0.93 (0.87–1.00)
2	78	3415	5.70	0.91 (0.85–0.98)
3	74	2771	5.60	0.91 (0.85–0.98)
4	68	2260	5.75	0.92 (0.85–0.99)
5 (most common)	57	1468	8.11	1.00 (ref)

a The incidence rate measures the number of new cases per 10 000 person-years at risk.

b Adjusted for age, gender, calendar period, parental psychiatric history and neighbourhood urbanicity (quintile) at age 15.

There was a slightly less marked reduction in the associated rate of psychosis in areas where these indices were less common when compared with area differences for those without these disadvantages. For those from a single-parent background only this difference was statistically significant, although only for the lowest quintile.

## Discussion

For each of the four examples of disadvantaged status examined we found no evidence of a positive association between being in a minority in a neighbourhood due to disadvantage and subsequent rates of psychosis. There was some evidence that a reduction in overall rates of psychosis associated with living in a less disadvantaged area was less marked for those who were disadvantaged, but this difference was small and, in most instances, not statistically significant.

### Strengths and limitations

This study had some important strengths. First, we were able to use interlinked national registry data collected over a period of several decades to thereby provide a wealth of information about the social circumstances of the cohort members. This allowed us to examine a range of different indices of disadvantage, at the individual and neighbourhood levels, with the exposure measured in advance of psychosis incidence thereby precluding reverse causality.

The study did, however, have some limitations. Although the longitudinal design meant that the area-level exposure information could not be directly influenced by the outcome of interest, it is still possible that an underlying genetic predisposition may have influenced where parents of cohort members chose to live. However, adjusting for secondary care treated mental health problems among parents made negligible difference to the observed results, thus implying that any residual effect would also have a negligible influence. It is also important to bear in mind that our study's findings may not be generalisable to other countries. For example, some types of inequality, such as income inequality, are less prevalent in Denmark (Causa, Hermansen, Ruiz, Klein, & Smidova, 2016). Therefore, the absence of a density effect according to income may, at least in part, be a reflection of Danish society. We also acknowledge the limitations of any register-based study where data collection is outside of the control of researchers (Thygesen & Ersbøll, 2014). Finally, it is worth stressing that, while we were able to look at a range of types of socioeconomic disadvantage, this list is far from exhaustive.

### Comparison with previous studies

We hypothesised that a similar relationship to that already shown for neighbourhood ethnic density would be seen for other types of disadvantages. Our previous study, using the same methodology as utilised in the present study, looked at a range of ethnic groups in Denmark and found that neighbourhood ethnic density, at age 15, was inversely associated with subsequent psychosis incidence for each group. For example, for those of African origin there was a 1.94-fold increase in non-affective psychosis (95% CI 1.17–3.23) comparing lowest and highest quintiles and for migrants from the Middle East we found a 1.68-fold (95% CI 1.19–2.38) increase. This is comparable with other ethnic density studies looking at different minority ethnic groups in different national contexts (Bécares et al., 2009; Boydell et al., 2001; Kirkbride et al., 2007; Veling et al., 2008b).

Comparing ethnic density with other group density effects begs the question: are these phenomena directly comparable? In terms of neighbourhood level variability, the indices that we examined are comparable, with similarly wide variation in the prevalence of each type of disadvantage compared to that reported for ethnic density in previous studies (Bécares et al., 2009; Boydell et al., 2001; Das-Munshi et al., 2010). For example, in one UK study reporting a significant ethnic density effect on rates of common mental disorders for people of Bangladeshi origin, the interquartile range for Bangladeshi ethnic density was between 11% and 47%. This is comparable to the inter-quintile range for density measures in our study (Table 1).

There have been few comparable studies of group density effects other than those investigating neighbourhood ethnic density. The nearest comparable study to ours assessed a set of similar cross-level interactions using a longitudinal design with school as a proxy for neighbourhood (Zammit et al., 2010). This Swedish population study looked at comparable exposures, measured at aged 16, although their study investigated a much earlier period, with the latest exposure occurring in 1993. They reported cross-level interactions between individual- and school-level ethnicity, social fragmentation and deprivation associated with psychosis incidence. Social fragmentation was measured using an index based on: immigration during childhood, recent internal migration and single-parent family status. Direct comparison with our measure based on single-parent family background is difficult as it is not possible to distinguish score components. Furthermore, the index applied in the earlier Swedish study is partly based on immigration, making it difficult to distinguish this from the effect of ethnic density. They also report a cross-level interaction using an index of deprivation, although the reported *p* value (0.06) is just beyond the standard statistical significance threshold. This finding is in contrast with our study, which found no evidence for a similar interaction effect for any of the examined markers of material deprivation.

Looking at previous cross-sectional studies, in our community survey study in South East London we found that being in a disadvantaged social class in a neighbourhood where this was less usual was unrelated to psychotic experiences (Schofield et al., 2016). However, we found living alone where this was less usual was associated with increased risk, as did an earlier study in Maastricht showing a similar association with likelihood of developing schizophrenia (van Os, Driessen, Gunther, & Delespaul, 2000). Other cross-sectional studies report similarly mixed results for other mental health outcomes. One study reports higher suicide rates for unemployed people living in low unemployment areas (Platt, 1986) while a Danish population register study using longitudinal data reported no interaction between individual and area socioeconomic characteristics and suicide rates (Agerbo et al., 2007). Also a recent study of depression prevalence among those in a minority due to low socioeconomic status failed to find any association with group density (Albor et al., 2014).

### Interpretation

The absence of evidence of group density effects in this study, in contrast with our previous ethnic density analyses, would seem to imply that group density effects are specific to ethnicity. However, we cannot infer evidence of absence from the absence of evidence (Altman & Bland, 1995) and, had we been able examine other indices of disadvantage, these may have revealed comparable group density effects. It is also possible that the ethnic group provides a better marker of an enduring ‘fundamental cause’ (Phelan, Link, & Tehranifar, 2010) of health inequality in comparison with the other socioeconomic measures in our study, which are all based on parental circumstances when the cohort member was 15 years old. However, the weight of current evidence suggests that ethnically defined group density is clearly related to psychosis risk, whereas studies of other examples of group density have, thus far, failed to show a comparable effect.

Why might this be the case? This may reflect a greater baseline risk of psychosis among ethnic minority groups compared to other disadvantaged groups. As a recent umbrella review concluded, ethnic group is the only socio-demographic factor shown to have a ‘convincing’ level of association (Radua et al., 2018) with the risk of psychosis whereas other examples of socioeconomic determinants show only weak or no evidence of association. Although different examples of social adversity appear to be important it is far from clear that they are consistently observed as risk factors. It may also be the case that ethnicity is simply a much stronger indicator of underlying risk factors, such as social status, than the other indicators we looked at. Ideally, a survey or qualitative approach is needed to examine this in more depth. However, it is notable that in our analysis we found very little suggestion that any other indicators showed a density effect, despite their potential relevance to underlying social status.

As we said in the Introduction, explanations for ethnic density effects can be grouped into those that prioritise racism and discrimination as relevant factors and more general explanations, such as those citing social support. If group density effects were specific to ethnicity, as our results appear to suggest, then this may then be in accordance with explanations proposed for the buffering effect of ethnic density against the pathogenic influence of racism and ethnic discrimination (Becares et al., 2013; Bécares et al., 2009; Das-Munshi et al., 2010; 2012). As a recent systematic review shows, perceived ethnic discrimination contributes to the higher prevalence of psychotic symptoms and experiences in ethnic minority groups (Bardol et al., 2020). Although evidence for a direct link with ethnic density has not yet been shown, studies have shown that perceived discrimination is negatively correlated with ethnic density (Bécares et al., 2009; Das-Munshi et al., 2012; Veling et al., 2008a).

Given a higher level of underlying psychosis risk, the protective benefits of increased social support conferred by living in an area with a much higher own-group density may still be more acute for minority ethnic groups, and the consequences of discriminatory practices and marginalisation therefore more likely to lead to ‘social defeat’, and subsequent psychosis (Selten & Cantor-Graae, 2005). What is notable in our study is how little these group density effects seem to apply to other disadvantaged groups.

Given this field of research is dominated by large-scale quantitative analyses there may also be important epistemological reasons for ethnic density appearing to be the defining group density effect. Although ethnic categories in quantitative analyses are typically crudely defined, by necessity to achieve statistical power, there are similar challenges with other attempts to determine social position. It may therefore be that ethnicity and migrant group, as recorded in electronic health records and register data, are simply better markers of social grouping when compared with other indicators of social position. Given that these are all predefined categories of social position it is possible that in-depth qualitative analysis may uncover the examples of lack of fit with the social environment that are currently beyond the reach of studies based on large-scale quantitative analysis.

There is still therefore much to learn about the relation between group density and psychosis and further longitudinal and in-depth qualitative studies, looking at other examples of group density in different national contexts, are now needed.

## Supplementary material

For supplementary material accompanying this paper visit https://doi.org/10.1017/S0033291721002233. click here to view supplementary material

## References

[ref1] Agerbo, E., Sterne, J. A. C., & Gunnell, D. J. (2007). Combining individual and ecological data to determine compositional and contextual socio-economic risk factors for suicide. Social Science & Medicine, 64, 451–461. 17050054 10.1016/j.socscimed.2006.08.043

[ref2] Albor, C., Uphoff, E. P., Stafford, M., Ballas, D., Wilkinson, R. G., & Pickett, K. E. (2014). The effects of socioeconomic incongruity in the neighbourhood on social support, self-esteem and mental health in England. Social Science & Medicine *(*1982*)*, 111, 1–9. 24735720 10.1016/j.socscimed.2014.04.002

[ref3] Altman, D. G., & Bland, J. M. (1995). Statistics notes: Absence of evidence is not evidence of absence. British Medical Journal, 311, 485. 7647644 10.1136/bmj.311.7003.485 PMC2550545

[ref4] Bardol, O., Grot, S., Oh, H., Poulet, E., Zeroug-Vial, H., Brunelin, J., & Leaune, E. (2020). Perceived ethnic discrimination as a risk factor for psychotic symptoms: A systematic review and meta-analysis. Psychological Medicine, 50, 1077–1089. 32317042 10.1017/S003329172000094X

[ref5] Becares, L., Cormack, D., & Harris, R. (2013). Ethnic density and area deprivation: Neighbourhood effects on Maori health and racial discrimination in Aotearoa/New Zealand. Social Science & Medicine, 88, 76–82. 23702212 10.1016/j.socscimed.2013.04.007 PMC3725420

[ref6] Bécares, L., Dewey, M. E., & Das-Munshi, J. (2018). Ethnic density effects for adult mental health: Systematic review and meta-analysis of international studies. Psychological Medicine, 48, 2054–2072. 29239292 10.1017/S0033291717003580 PMC6076993

[ref7] Bécares, L., Nazroo, J., Stafford, M., Becares, L., Nazroo, J., & Stafford, M. (2009). The buffering effects of ethnic density on experienced racism and health. Health and Place, 15, 670–678. 19117792 10.1016/j.healthplace.2008.10.008

[ref8] Bourdieu, P. (1984). Distinction: A Critique of the social judgement of Taste. Cambridge: Harvard University Press.

[ref9] Bourque, F., van der Ven, E., & Malla, A. (2011). A meta-analysis of the risk for psychotic disorders among first- and second-generation immigrants. Psychological Medicine, 41, 897–910. 20663257 10.1017/S0033291710001406

[ref10] Boydell, J., van Os, J., McKenzie, K., Allardyce, J., Goel, R., McCreadie, R. G., & Murray, R. M. (2001). Incidence of schizophrenia in ethnic minorities in London: Ecological study into interactions with environment. BMJ, 323, 1336–1338. 11739218 10.1136/bmj.323.7325.1336 PMC60671

[ref11] Breslow, N. E. (1984). Extra-Poisson variation in log-linear models. Applied Statistics, 33, 38–44.

[ref12] Byrne, M., Agerbo, E., Eaton, W. W., & Mortensen, P. B. (2004). Parental socio-economic status and risk of first admission with schizophrenia – A Danish national register based study. Social Psychiatry and Psychiatric Epidemiology, 39, 87–96. 15052389 10.1007/s00127-004-0715-y

[ref13] Cantor-Graae, E., & Selten, J. P. (2005). Schizophrenia and migration: A meta-analysis and review. American Journal of Psychiatry, 162, 12–24. 15625195 10.1176/appi.ajp.162.1.12

[ref14] Carstensen, B. (2007). Age-period-cohort models for the Lexis diagram. Statistics in Medicine, 26, 3018–3045. 17177166 10.1002/sim.2764

[ref15] Causa, O., Hermansen, M., Ruiz, N., Klein, C., & Smidova, Z. (2016). Inequality in Denmark through the looking Glass. OECD Economics Department Working Papers no. 1341.

[ref16] Corcoran, C., Perrin, M., Harlap, S., Deutsch, L., Fennig, S., Manor, O., … Susser, E. (2009). Incidence of Schizophrenia among second-generation immigrants in the Jerusalem perinatal cohort. Schizophrenia Bulletin, 35, 596–602. 18648022 10.1093/schbul/sbn089 PMC2669576

[ref17] Das-Munshi, J., Bécares, L., Boydell, J. E., Dewey, M. E., Morgan, C., Stansfeld, S. A., & Prince, M. J. (2012). Ethnic density as a buffer for psychotic experiences: Findings from a national survey (EMPIRIC). The British Journal of Psychiatry: The Journal of Mental Science, 201, 282–290. 22844021 10.1192/bjp.bp.111.102376 PMC3461446

[ref18] Das-Munshi, J., Becares, L., Dewey, M. E., Stansfeld, S. A., & Prince, M. J. (2010). Understanding the effect of ethnic density on mental health: Multi-level investigation of survey data from England. BMJ, 341, c5367–c5367. 20966012 10.1136/bmj.c5367 PMC2962884

[ref19] Dohrenwend, B. P., Levav, I., Shrout, P. E., Schwartz, S., Naveh, G., Link, B. G., … Stueve, A. (1992). Socioeconomic status and psychiatric disorders: The causation-selection issue. Science (New York, N.Y.), 255, 946–952. 1546291 10.1126/science.1546291

[ref20] Eaton, W. W. (1974). Residence, social class, and schizophrenia. Journal of Health and Social Behavior, 15, 289–299. 4455732

[ref21] Gevonden, M. J., Selten, J. P., Myin-Germeys, I., De Graaf, R., Ten Have, M., Van Dorsselaer, S., … Veling, W. (2014). Sexual minority status and psychotic symptoms: Findings from the Netherlands Mental Health Survey and Incidence Studies (NEMESIS). Psychological Medicine, 44, 421–433. 23710972 10.1017/S0033291713000718

[ref22] Hakulinen, C., Webb, R. T., Pedersen, C. B., Agerbo, E., & Mok, P. L. H. (2020). Association between parental income during childhood and risk of schizophrenia later in life. American Medical Association JAMA Psychiatry, 77, 17–24. 10.1001/jamapsychiatry.2019.2299 PMC6813592 31642886

[ref23] Halpern, D. (1993). Minorities and mental health. Social Science and Medicine, 36, 597–607. 8456329 10.1016/0277-9536(93)90056-a

[ref24] Hatzenbuehler, M. L., Keyes, K. M., & McLaughlin, K. A. (2011). The protective effects of social/contextual factors on psychiatric morbidity in LGB populations. International Journal of Epidemiology, 40, 1071–1080. 21330343 10.1093/ije/dyr019 PMC3156367

[ref25] Kirkbride, J. B., Morgan, C., Fearon, P., Dazzan, P., Murray, R., & Jones, P. B. (2007a). Neighbourhood-level effects on psychoses: Re-examining the role of context. Psychological Medicine, 37, 1413–1425. 17472758 10.1017/S0033291707000499

[ref26] Krabbendam, L., & van Os, J. (2005). Schizophrenia and urbanicity: A major environmental influence – Conditional on genetic risk. Schizophrenia Bulletin, 31, 795–799. 16150958 10.1093/schbul/sbi060

[ref27] Marwaha, S., Johnson, S., Bebbington, P., Stafford, M., Angermeyer, M. C., Brugha, T., … Toumi, M. (2007). Rates and correlates of employment in people with schizophrenia in the UK, France and Germany. British Journal of Psychiatry, 191, 30–37. 10.1192/bjp.bp.105.020982 17602122

[ref28] Meltzer, H., Singleton, N., Lee, A., Bebbington, P., Brugha, T., & Jenkins, R. (2002). The social and economic circumstances of adults with mental disorders. London: The Stationery Office.

[ref29] Morgan, C., Kirkbride, J., Hutchinson, G., Craig, T., Morgan, K., Dazzan, P., … Fearon, P. (2008). Cumulative social disadvantage, ethnicity and first-episode psychosis: A case-control study. Psychological Medicine, 38, 1701. 19000327 10.1017/S0033291708004534

[ref30] Mors, O., Perto, G. P., & Mortensen, P. B. (2011). The Danish psychiatric central research register. Scandinavian Journal of Public Health, 39, 54–57. 21775352 10.1177/1403494810395825

[ref31] Muntaner, C., Tien, A. Y., Eaton, W. W., & Garrison, R. (1991). Occupational characteristics and the occurrence of psychotic disorders. Social Psychiatry and Psychiatric Epidemiology, 26, 273–280. 1792558 10.1007/BF00789219

[ref32] Pedersen, C. B. (2006). No evidence of time trends in the urban–rural differences in schizophrenia risk among five million people born in Denmark from 1910 to 1986. Psychological Medicine, 36, 211–219. 16303060 10.1017/S003329170500663X

[ref33] Pedersen, C. B. (2011). The Danish civil registration system. Scandinavian Journal of Public Health, 39, 22–25. 21775345 10.1177/1403494810387965

[ref34] Pedersen, C. B., & Mortensen, P. B. (2001). Evidence of a dose-response relationship between urbanicity during upbringing and schizophrenia risk. Archives of General Psychiatry, 58, 1039–1046. 11695950 10.1001/archpsyc.58.11.1039

[ref35] Phelan, J. C., Link, B. G., & Tehranifar, P. (2010). Social conditions as fundamental causes of health inequalities: Theory, evidence, and policy implications. Journal of Health and Social Behavior, 51, S28–S40. 20943581 10.1177/0022146510383498

[ref36] Platt, S. (1986). Parasuicide and unemployment. British Journal of Psychiatry, 149, 401–405. 10.1192/bjp.149.4.401 3545352

[ref37] Radua, J., Ramella-Cravaro, V., Ioannidis, J. P. A., Reichenberg, A., Phiphopthatsanee, N., Amir, T., … Fusar-Poli, P. (2018). What causes psychosis? An umbrella review of risk and protective factors. World Psychiatry, 17, 49–66. 29352556 10.1002/wps.20490 PMC5775150

[ref38] Rosenberg, M. (1962). The dissonant religious context and emotional disturbance. American Journal of Sociology of Health and Illness, 10, 1–10.

[ref39] Schofield, P., Das-Munshi, J., Becares, L., Morgan, C., Bhavsar, V., Hotopf, M., & Hatch, S. L. (2016). Minority status and mental distress: A comparison of group density effects. Psychological Medicine, 46, 3051–3059. 27523979 10.1017/S0033291716001835 PMC5080664

[ref40] Schofield, P., Thygesen, M., Das-Munshi, J., Becares, L., Cantor-Graae, E., Agerbo, E., … Pedersen, C. (2018). Neighbourhood ethnic density and psychosis – Is there a difference according to generation? Elsevier B.V. Schizophrenia Research, 22, 965–973. 10.1016/j.schres.2017.09.029 PMC5889713 28969931

[ref41] Schofield, P., Thygesen, M., Das-Munshi, J., Becares, L., Cantor-Graae, E., Pedersen, C., … Agerbo, E. (2017). Ethnic density, urbanicity and psychosis risk for migrant groups – A population cohort study. Schizophrenia Research, 190, 82–87. 28318842 10.1016/j.schres.2017.03.032 PMC5735221

[ref42] Selten, J. P., & Cantor-Graae, E. (2005). Social defeat: Risk factor for schizophrenia? British Journal of Psychiatry, 191, 9–12. 10.1192/bjp.187.2.101 16055818

[ref43] Selten, J. P., Van Der Ven, E., & Termorshuizen, F. (2019). Migration and psychosis: A meta-analysis of incidence studies. Psychological Medicine, 50, 303–313. 30722795 10.1017/S0033291719000035 PMC7083571

[ref44] Selten, J. P., Veen, N., Feller, W., Blom, J. D., Schols, D., Camoenië, W., … Kahn, R. (2001). Incidence of psychotic disorders in immigrant groups to the Netherlands. British Journal of Psychiatry, 178, 367–372. 10.1192/bjp.178.4.367 11282817

[ref45] Shaw, R. J., Atkin, K., Bécares, L., Albor, C. B., Stafford, M., Kiernan, K. E., … Pickett, K. E. (2012). Impact of ethnic density on adult mental disorders: Narrative review. British Journal of Psychiatry, 201, 11–19. 10.1192/bjp.bp.110.083675 22753852

[ref46] Thygesen, L. C., & Ersbøll, A. K. (2014). When the entire population is the sample: Strengths and limitations in register-based epidemiology. European Journal of Epidemiology, 29, 551–558. 24407880 10.1007/s10654-013-9873-0

[ref47] van Os, J., Driessen, G., Gunther, N., & Delespaul, P. (2000). Neighbourhood variation in incidence of schizophrenia. Evidence for person-environment interaction. British Journal of Psychiatry, 176, 243–248. 10.1192/bjp.176.3.243 10755071

[ref48] Vassos, E., Pedersen, C. B., Murray, R. M., Collier, D. A., & Lewis, C. M. (2012). Meta-analysis of the association of urbanicity with schizophrenia. Schizophrenia Bulletin, 38, 1118–1123. 23015685 10.1093/schbul/sbs096 PMC3494055

[ref49] Veling, W., Hoek, H. W., & Mackenbach, J. P. (2008a). Perceived discrimination and the risk of schizophrenia in ethnic minorities. Social Psychiatry and Psychiatric Epidemiology, 43, 953–959. 18575790 10.1007/s00127-008-0381-6

[ref50] Veling, W., Susser, E., van Os, J., Mackenbach, J. P., Selten, J.-P., & Hoek, H. W. (2008b). Ethnic density of neighborhoods and incidence of psychotic disorders among immigrants. American Journal of Psychiatry, 165, 66–73. 18086750 10.1176/appi.ajp.2007.07030423

[ref51] Wechsler, H., & Pugh, T. F. (1967). Fit of individual and community characteristics and rates of psychiatric hospitalization. American Journal of Sociology, 73, 331–338.

[ref52] Werner, S., Malaspina, D., & Rabinowitz, J. (2007). Socioeconomic status at birth is associated with risk of schizophrenia: Population-based multilevel study. Schizophrenia Bulletin, 33, 1373–1378. 17443013 10.1093/schbul/sbm032 PMC2779876

[ref53] Zammit, S., Lewis, G., Rasbash, J., Dalman, C., Gustafsson, J. E., & Allebeck, P. (2010). Individuals, schools, and neighborhood: A multilevel longitudinal study of variation in incidence of psychotic disorders. Archives of General Psychiatry, 67, 914–922. 20819985 10.1001/archgenpsychiatry.2010.101

