# Supplementary material for: Lack of fit with the neighbourhood social environment as a risk factor for psychosis – a national cohort study
Source: Psychol Med. 2021 Jun 18;53(3):866–74. doi: 10.1017/S0033291721002233 (PMC9975998; doi:10.1017/S0033291721002233)
Supplement: Supplementary file 1 [file S0033291721002233sup001.docx]

## Appendix

Table 4 Missing data for individual and area level predictors

|  | Missing data (%) | |
| --- | --- | --- |
|  | Individual level (Parental status at age 15) | Area level  (Parish at age 15) |
| Not employed | 4.0 | 4.7 |
| Manual occupation | 3.9 | 0.6 |
| Single parent | 1.3 | - ^a^ |
| Low income | 2.1 | 5.6 ^b^ |

^a^ a record of missing data for single parents at area level was not collected

^b^ based on aggregated data for the cohort members’ peer group only

Table 5 Incidence rate ratios of non-affective psychosis by neighbourhood congruency (based on neighbourhood profile at age 15) for cohort members with each type of disadvantaged status – continuous exposure variable model

| Disadvantaged status (parent circumstances at age 15) and corresponding neighbourhood profile | Average (mean) neighbourhood percentage with corresponding disadvantaged status (%) | Cases | Crude Incidence Rate^a^ | Incidence rate ratios (95% CI) corresponding to a 10% reduction in neighbourhood percentage with corresponding disadvantaged status – fully adjusted model^b^ |
| --- | --- | --- | --- | --- |
| Not employed | 20 | 4,253 | 13.54 | 1.02 (0.97 - 1.07) |
| Manual occupation | 34 | 2,595 | 13.96 | 0.91 (0.86 - 0.96) |
| Single parent | 23 | 6,953 | 13.17 | 0.92 (0.89 - 0.95) |
| Low income | 24 | 8,316 | 11.13 | 1.01 (0.99 - 1.04) |

^a^The incidence rate measures the number of new cases per 10,000 person-years at risk

^b^Adjusted for age, gender calendar period, parental psychiatric history and neighbourhood urbanicity (quintile) at age 15.

Table 6 Incidence rate ratios of non-affective psychosis by neighbourhood congruency (based on neighbourhood profile at age 15) for cohort members without the corresponding disadvantaged status – using continuous measure of area exposure

| Disadvantaged status *not present* (parent circumstances at age 15) and corresponding neighbourhood profile | Average (mean) neighbourhood percentage who do not have the corresponding disadvantaged status (%) | Cases | Crude Incidence Rate^a^ | Incidence rate ratios (95% CI) corresponding to a 10% reduction in neighbourhood percentage without corresponding disadvantaged status – fully adjusted model^b^ |
| --- | --- | --- | --- | --- |
| Not employed | 80 | 18,442 | 6.30 | 0.95 (0.92 - 0.98) |
| Manual occupation | 66 | 7,141 | 8.08 | 0.90 (0.86 - 0.94) |
| Single parent | 77 | 16,920 | 6.03 | 0.86 (0.84 - 0.89) |
| Low income | 76 | 15,224 | 5.94 | 0.99 (0.97 - 1.01) |

^a^The incidence rate measures the number of new cases per 10,000 person-years at risk

^b^Adjusted for age, gender calendar period, parental psychiatric history and neighbourhood urbanicity (quintile) at age 15.

Table 7 Sensitivity analysis - incidence rate ratios of non-affective psychosis by neighbourhood congruency (table 2) further adjusted for area level income

| Disadvantaged status (parent circumstances at age 15) and corresponding neighbourhood profile (quintiles) | Incidence rate ratios (95% CI) – further adjusted model^a^ |
| --- | --- |
| Not employed |  |
| 1 (least common) | 1.00 (0.89 - 1.12) |
| 2 | 1.11 (0.99 - 1.23) |
| 3 | 1.04 (0.93 - 1.16) |
| 4 | 1.00 (0.90 - 1.11) |
| 5 (most common) | 1.00 (ref) |
| Manual occupation |  |
| 1 (least common) | 0.90 (0.77 - 1.06) |
| 2 | 0.86 (0.74 - 1.00) |
| 3 | 0.99 (0.85 - 1.15) |
| 4 | 1.04 (0.89 - 1.21) |
| 5 (most common) | 1.00 (ref) |
| Single parent |  |
| 1 (least common) | 0.76 (0.68 - 0.85) |
| 2 | 0.78 (0.71 - 0.87) |
| 3 | 0.86 (0.78 - 0.94) |
| 4 | 0.89 (0.82 - 0.97) |
| 5 (most common) | 1.00 (ref) |

^a^Adjusted for age, gender calendar period, parental psychiatric history, neighbourhood urbanicity (quintile) and neighbourhood low income (quintile) at age 15.
